# Identifying the Novel Gut Microbial Metabolite Contributing to Metabolic Syndrome in Children Based on Integrative Analyses of Microbiome-Metabolome Signatures

**DOI:** 10.1128/spectrum.03771-22

**Published:** 2023-02-16

**Authors:** Jia Wei, Wen Dai, Xiongfeng Pan, Yan Zhong, Ningan Xu, Ping Ye, Jie Wang, Jina Li, Fei Yang, Jiayou Luo, Miyang Luo

**Affiliations:** a Xiangya School of Public Health, Central South University, Changsha, Hunan, China; b Hunan Provincial Key Laboratory of Clinical Epidemiology, Central South University, Changsha, Hunan, China; c Institute of Children Health, Hunan Children’s Hospital, Changsha, Hunan, China; d Hunan Province Key Laboratory of Typical Environmental Pollution and Health Hazards, School of Public Health, University of South China, Hengyang, Hunan, China

**Keywords:** metabolic syndrome, children, microbiota, metabolites, lipid metabolism, inflammation

## Abstract

The pathogenesis of gut microbiota and their metabolites in the development of metabolic syndrome (MS) remains unclear. This study aimed to evaluate the signatures of gut microbiota and metabolites as well as their functions in obese children with MS. A case-control study was conducted based on 23 MS children and 31 obese controls. The gut microbiome and metabolome were measured using 16S rRNA gene amplicon sequencing and liquid chromatography-mass spectrometry. An integrative analysis was conducted, combining the results of the gut microbiome and metabolome with extensive clinical indicators. The biological functions of the candidate microbial metabolites were validated *in vitro*. We identified 9 microbiota and 26 metabolites that were significantly different from the MS and the control group. The clinical indicators of MS were correlated with the altered microbiota *Lachnoclostridium*, *Dialister*, and *Bacteroides*, as well as with the altered metabolites all-trans-13,14-dihydroretinol, DL-dipalmitoylphosphatidylcholine (DPPC), LPC 24: 1, PC (14:1e/10:0), and 4-phenyl-3-buten-2-one, etc. The association network analysis further identified three MS-linked metabolites, including all-trans-13,14-dihydroretinol, DPPC, and 4-phenyl-3-buten-2-one, that were significantly correlated with the altered microbiota. Bio-functional validation showed that all-trans-13, 14-dihydroretinol could significantly upregulate the expression of lipid synthesis genes and inflammatory genes. This study identified a new biomarker that may contribute to MS development. These findings provided new insights regarding the development of efficient therapeutic strategies for MS.

**IMPORTANCE** Metabolic syndrome (MS) has become a health concern worldwide. Gut microbiota and metabolites play an important role in human health. We first endeavored to comprehensively analyze the microbiome and metabolome signatures in obese children and found the novel microbial metabolites in MS. We further validated the biological functions of the metabolites *in vitro* and illustrated the effects of the microbial metabolites on lipid synthesis and inflammation. The microbial metabolite all-trans-13, 14-dihydroretinol may be a new biomarker in the pathogenesis of MS, especially in obese children. These findings were not available in previous studies, and they provide new insights regarding the management of metabolic syndrome.

## INTRODUCTION

Metabolic syndrome (MS) refers to a cluster of symptoms, including central obesity, hypertension, and dyslipidemia (1). It is a typical metabolic disease in obese children, and the prevalence of MS is growing worldwide, with approximately 39% of obese children being predicted to have the disease (2). Moreover, the obese children without a metabolic disorder might be in a temporary status that could eventually develop into metabolic syndrome (3, 4). Currently, no effective pharmacological therapy is available to manage all of the metabolic abnormalities of MS (5). Moreover, untreated MS also increased the risk of cardiovascular disease (CVD), type 2 diabetes mellitus (T2DM), metabolic associated fatty liver disease (MAFLD), and certain types of cancers, which brought huge economic burdens to individuals and society (6–8). Therefore, it is urgent to comprehensively elucidate the pathogenesis of MS and to identify new biomarkers or targets associated with MS in children.

Recent studies have revealed the critical role of gut microbiota in the development of MS. Several gut microbiota that are associated with MS in adults were identified. For instance, the genera Lachnospira, Coprococcus, Ruminococcus, and Streptococcus were significantly enriched in MS adults, whereas Bacteroides, Roseburia, and Alistipes were enriched in controls that were without MS (9–11). However, obesity was linked to gut dysbiosis and probably influenced the association of gut microbiota and MS (12–14). Some studies have explored the different gut microbiota between MS adults and obese controls, and they have reported that the relative abundance of the genera Succinivibrio, Romboutsia, Terrisporobacter, and Eggerthia were enriched in obese controls, whereas the genera Faecalibacterium, Parabacteroides, Alistipes, Escherichia-Shigella, and Megamonas were enriched in the MS patients (15, 16). It was obvious that the different gut microbiota profiles in the MS patients and the obese controls were different from those of the MS patients and normal weight controls. Moreover, the gut microbiota relating to MS in children might differ from those of adults (15). However, few studies have investigated the gut microbiota of children in relation to MS, especially regarding the differences in gut microbiota between obese children with MS and obese controls. Hence, it is important to identify the specific gut microbiota that are associated with MS in Chinese children.

To our knowledge, the microbial composition not only shapes the environment of the gastrointestinal tract directly but also mediates the host metabolism through diverse metabolites. Growing evidence suggests that microbial metabolites, including short-chain fatty acids (SCFAs), branched-chain amino acids (BCAA), trimethylamine-N-oxide (TMAO), and bile acids (BAs) are implicated in the pathogenesis of metabolic diseases, such as MAFLD, CVD, and T2DM (17–19). In addition, a single metabolomics study derived the association of the circulating metabolites with MS among children who were 5 years of age, but the association of metabolites and gut microbiota was still unclear (20). To date, no research has revealed the association of microbial metabolites with MS in either adults or children.

Furthermore, exploring the interactions between disease-linked microbiota and metabolites may provide further insights into the pathogenic mechanisms of metabolic diseases. On one hand, previous studies have developed several prediction models for metabolic diseases using gut microbial and metabolites, based on multiomics data; however, these studies failed to investigate the biological functions and molecular mechanisms of these biomarkers in disease development (21–23). On the other hand, some research investigated the essential role of metabolites in disease progression, but it remained unclear whether these metabolites were derived from gut microbiota (24, 25). Additionally, earlier studies focused exclusively on the alteration of gut microbiota or metabolite profiles that were linked with metabolic disorder, whereas the crosstalk among microbiota, metabolites, and the host metabolism was usually ignored.

Here, we aimed to analyze the gut microbiome and untargeted metabolome data of MS and obese children and perform the association network analysis for the gut microbiome, metabolome, and typical clinical indicators. Then, the biological functions of the candidate microbial metabolite were validated *in vitro*. This study might provide new insights into the development of novel potential therapeutic therapies for MS.

## RESULTS

### Characteristics of MS patients and obese controls.

54 children between 10 and 18 years of age were enrolled in this study, including 23 obese children with MS and 31 obese children without MS (the control group). The characteristics of these participants are presented in Table 1 and Table S1. No significant differences in age or gender composition were observed between the two groups. The systolic blood pressure (SBP) level in the MS patients was slightly higher than that observed in the controls. Compared with the controls, the levels of serum triglycerides (TG) and total cholesterol (CHOL) were significantly higher in children with MS. The levels of serum high-density lipoprotein cholesterol (HDL-c) were significantly lower in the MS group than those observed in the control group. Moreover, there were no significant differences in the other indicators between the two groups. The details are listed in Table S1.

**TABLE 1:** Characteristics of MS patients and obese subjects^*a*^

Characteristic	MS (*n* = 23)	Control (*n* = 31)	*P* value
Female gender (%)	95.65	96.77	0.652
Age (yr)	13.87 ± 1.91	13.26 ± 1.39	0.520
BMI (kg/m^2^)	28.84 ± 3.68	28.55 ± 3.48	0.900
wt (kg)	69.99 ± 16.75	66.03 ± 15.26	0.817
Body fat mass (kg)	26.14 ± 6.61	26.80 ± 8.51	0.295
Lean body mass (kg)	43.60 ± 11.50	40.12 ± 8.35	0.213
Percentage of body fat (%)	37.46 ± 4.78	39.77 ± 6.12	0.600
Visceral fat (cm^2^)	121.31 ± 28.80	131.64 ± 40.93	0.163
SBP (mm Hg)	128.31 ± 9.97	120.23 ± 11.61	0.018*
DBP (mm Hg)	71.87 ± 11.08	72.71 ± 11.67	0.790
TG (mmol/L)	2.09 ± 0.97	1.60 ± 0.27	<0.001*
CHOL (mmol/L)	4.24 ± 0.23	3.71 ± 0.24	0.012*
HDL-c (mmol/L)	1.03 ± 0.23	1.22 ± 0.152	0.001*
LDL-c (mmol/L)	2.33 ± 0.75	2.092 ± 0.62	0.381
ALB (g/L)	42.92 ± 1.90	41.23 ± 2.40	0.106
GLO (g/L)	29.50 ± 2.45	29.26 ± 3.02	0.118
FPG (mmol/L)	4.77 ± 1.89	4.32 ± 0.41	0.084
Insulin (μU/mL)	35.72 ± 20.78	29.87 ± 13.43	0.115
C-peptide (ng/mL)	3.99 ± 1.60	3.60 ± 1.03	0.073
AST (IU/L)	31.89 ± 18.33	28.82 ± 16.20	0.518
ALT (IU/L)	57.43 ± 10.34	52.64 ± 49.34	0.706
AST/ALT	0.62 ± 0.15	0.73 ± 0.303	0.007*

a Values are expressed as means ± SD. BMI, body mass index; SBP, systolic blood pressure; DBP, diastolic blood pressure; TG, triglycerides; CHOL, total cholesterol; HDL-c, high-density lipoprotein cholesterol; LDL-c, low-density lipoprotein cholesterol; FPG, fasting plasma glucose; AST, aspartate aminotransferase; ALT, alanine aminotransferase; ALB, albumin; GLO, globulin. *t* tests were used to evaluate the differences in anthropometric, demographic data and clinical laboratory data between the two groups. Unless otherwise stated, a *P* value of <0.05 (*) was considered to be indicative of a statistically significant result. MS: obese children with MS; Control: obese children.

### Gut microbiota signatures of MS children and controls.

**FIG 1:**
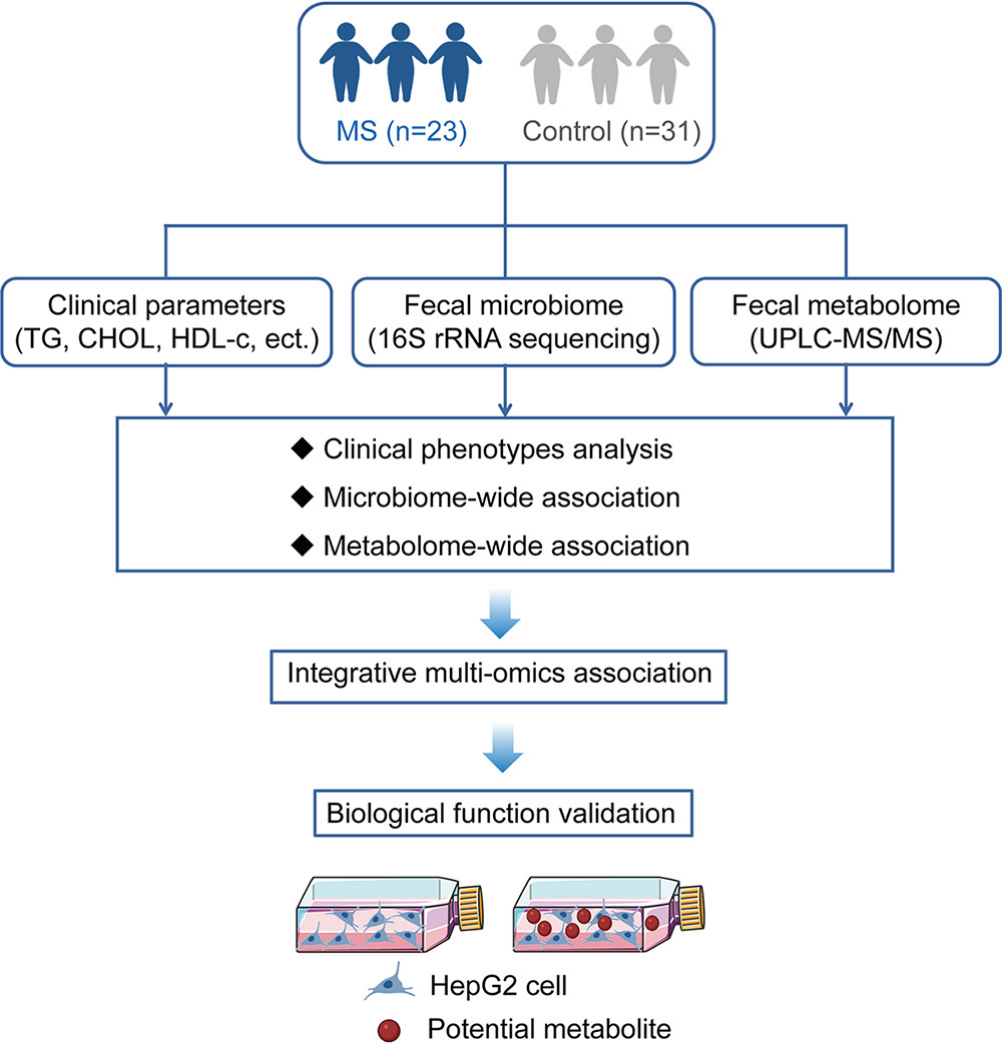
Overview of the study design.

The sequencing depth ranged from 44,679 to 67,693 counts per sample (mean = 59,919). The sequencing depth and quality control of each sample approached the expected level. The rarefaction curve is shown in Fig. S1, and it suggests that the estimated OTUs richness basically approached saturation in all samples (Fig. S1). The alpha-diversity values, including richness indices (Chao1 and ACE) and diversity indices (Shannon and Simpson) were compared between the MS and control groups, but no significant differences were observed (*P* > 0.05) (Fig. 1A; Fig. S2). A PCoA of the beta-diversity suggested that the MS group slightly deviated from the control group in terms of global community composition, but no significant difference was observed; only the plot of the PCoA, based on the Bray-Curtis distance, is shown (*P* > 0.05) (Fig. 1B). Overall, the composition of gut microbiota was similar in the MS group and the control group, but the relative abundance percentages of some taxa were different between the two groups. At the phylum level, *Firmicutes* was the most predominant bacterial phylum, accounting for 64.28% and 58.46% of the gut microbiota in the MS group and the control group, respectively. *Bacteroidetes* was the second most predominant phylum, accounting for 20.64% and 24.60% abundance in the MS group and the control group, respectively (Fig. 1C). Moreover, the MS group exhibited a significantly increased *Firmicutes*/*Bacteroidetes* (F/B) ratio (*P* < 0.01 and *P_fdr_* < 0.05) (Fig. 1D). At the genus level, *Blautia* (8.36%) and *unidentified_Lachnospiraceae* (7.84%) were enriched in the MS group, but *Bacteroides* (15.94%) and *Bifidobacterium* (5.99%) were enriched in the control group (Fig. 1E).

### Functional prediction and correlation of altered microbiota with clinical characteristics.

**FIG 2:**
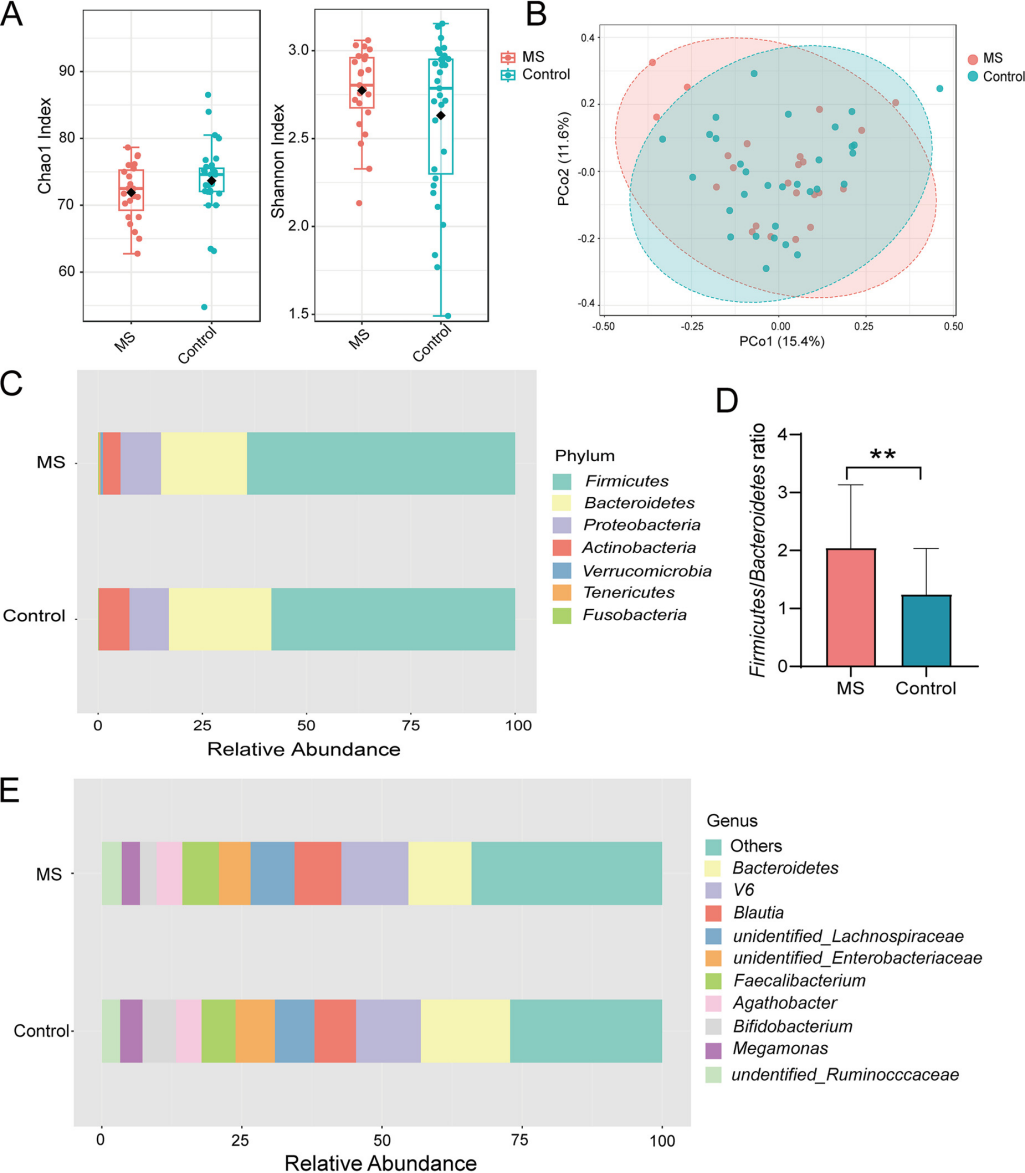
Microbiota diversity and composition in the fecal sample. (A) Alpha diversity between the MS and the control group. (B) Beta diversity indices in the MS and the control group. (C) The composition and relative abundance of gut microbiota at the phylum level. (D) The relative abundance of the F/B ratio in the MS and the control group. (E) The composition and relative abundance of gut microbiota at the genus level.

A linear discriminant analysis (LDA) effect size (LEfSe) analysis was used to determine the maximum difference in the microbial structure between the MS children and the controls, and this analysis identified 6 predominant microbial markers in the two groups. *Dialister*, *Anaerostipes*, and *Lachnoclostridium* exhibited higher abundance in the MS group, whereas the abundance of the genera *Bacteroides*, *Subdoligranulum*, and *Parabacteroides* were enriched in the control group (*P* < 0.01 and *P_fdr_* < 0.05) (Fig. 2A). The difference between these representative microbial markers at the genus level between the MS children and the control group were confirmed via further testing methods, and it remained significant (Fig. S3). The microbial functions were predicted so as to explore the functions of the gut microbiota that are involved in the development of MS. The HIF-1 signaling pathway and mineral absorption pathway were enriched in the MS group. Microbial metabolic pathways in the control group were more enriched in themogenesis and flavone flavonol biosynthesis (Fig. S4). However, these metabolic pathways were not significantly different between the MS group and the control group (*P* > 0.01). In order to identify the MS-linked microbiota, correlation analyses were performed between the significantly altered gut microbiota and typical clinical indicators. Fig. 2B shows the correlation heat map between the gut microbiota and the clinical indicators, and *Lachnoclostridium* is distinguished from the other five altered genera. The abundance of *Lachnoclostridium* was positively correlated with CHOL, FPG, and LDL-c levels (*P* < 0.01 and *P_fdr_* < 0.05). The abundance of *Bacteroides* was negatively correlated with the TG and CHOL levels (*P* < 0.05) (Fig. 2B). The *Dialister* abundance was positively correlated with the levels of DBP (*P* < 0.01 and *P_fdr_* < 0.05) (Fig. 2B). In addition, *Aneaerostipes*, *Parabacteroides*, and *Subdoligranulum* suggested no significant correlation with the MS clinical indicators.

### Gut metabolome alterations in the MS children and in the controls.

**FIG 3:**
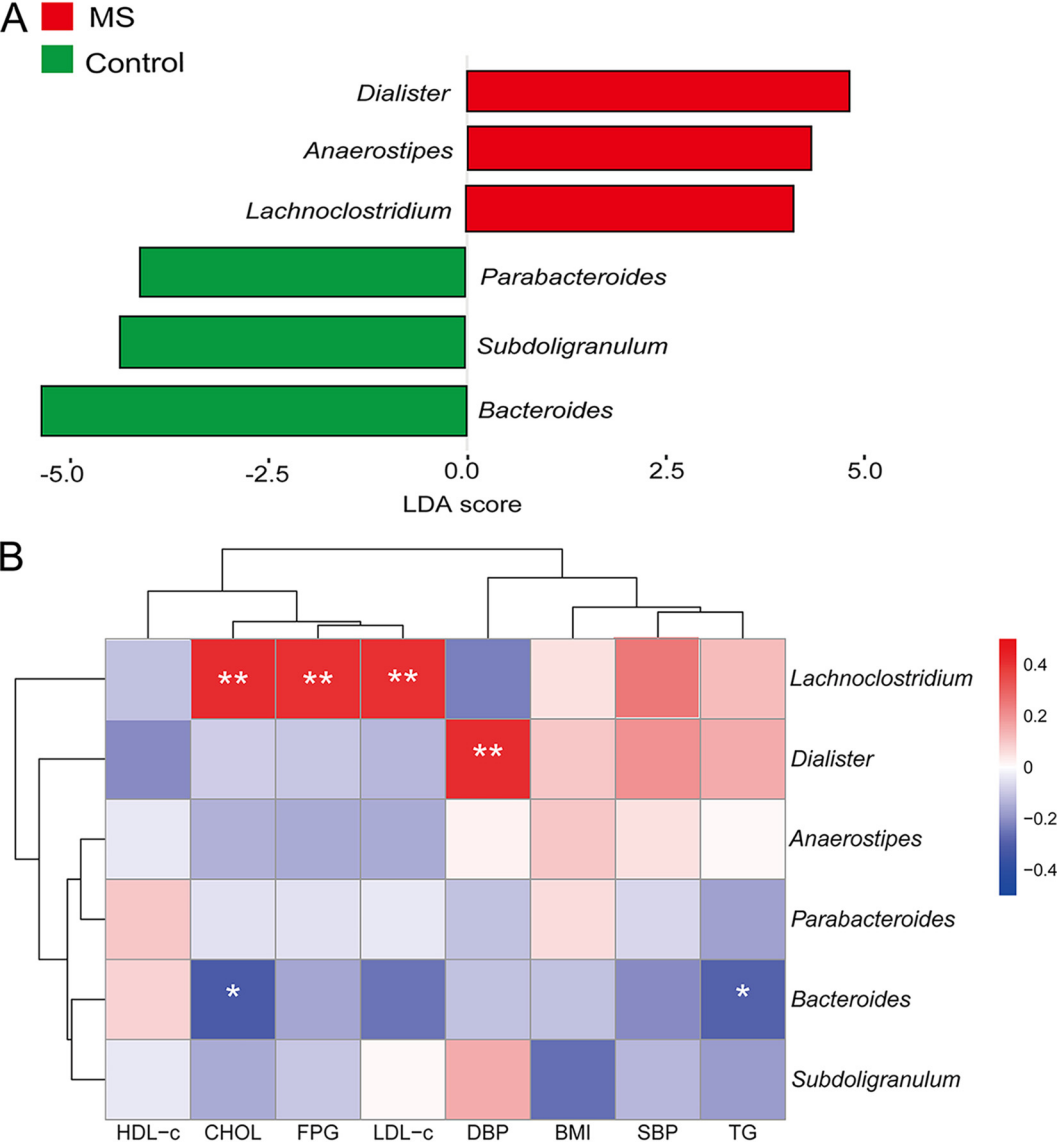
Gut microbiota analysis between MS children and obese controls. (A) A LEfSe analysis showed the taxa that significantly differed between groups. Taxa with an LDA score of >3 and a *p_fdr_* of <0.05 are shown. (B) Correlation heat map analysis between the gut microbiota and clinical indicators. Red represents a positive correlation, and blue represents a negative correlation. *, *P* < 0.05; **, *P* < 0.01.

Considering the interplay between the gut microbiome and the host metabolism, nontargeted metabolomic analyses were performed on fecal samples. A total of 2,491 gut metabolites were quantified. Partial least-squares discrimination analysis (PLS-DA) score plots revealed that the profiles of the metabolites were obviously separated between the MS and control groups (Fig. 3A). As shown in the Volcano plot (Fig. 3B), 29 metabolites were significantly altered when the MS group was compared with the control group (*P* < 0.05). Moreover, 26 out of 29 different metabolic fragments were structurally identified in the mass spectral library (Table S2). Among them, 16 metabolites were increased, whereas 10 metabolites were decreased, in the MS group. In particular, all-trans-13,14-dihydroretinol, vitamin A, 4-phenyl-3-buten-2-one, DL-dipalmitoylphosphatidylcholine (DPPC), and isotretinoin were significantly enriched in the MS group (*P* < 0.05 and *P_fdr_* < 0.05). In contrast, other metabolites, including PC (14:1e/10:0), PC (16:0/17:2), LPC 24:1, N-acetyl-d-glucosamine 6-phosphate (GlcNAc-6P), and trans-delta2-11-methyl-dodecenoic acid were depleted in the MS group, compared with the control group (*P* < 0.05 and *P_fdr_* < 0.05) (Fig. 3C).

### Pathway enrichment analysis and correlation of altered gut metabolites with clinical characteristics.

To understand the functions of the significantly altered gut metabolites in the MS and control groups, a pathway enrichment analysis was conducted using the Kyoto Encyclopedia of Genes and Genomes (KEGG) annotations. The metabolites involved in the retinol metabolism pathway and in the amino sugar and nucleotide sugar metabolism pathway were significantly enriched (*P* < 0.05) (Fig. 3D). Furthermore, a correlation analysis between the top 10 altered metabolites (*P* < 0.05 and *P_fdr_* < 0.05) and typical clinical indicators was performed to explore the associations of differential metabolites with MS progression (Fig. 3F; Table S3). Consistent with the results of the comparison between groups, all-trans-13, 14-dihydroretinol in the retinol metabolism pathway was positively correlated with the serum level of TG (*P* < 0.05) and negatively correlated with HDL-c (*P* < 0.05 and *P_fdr_* < 0.05) (Fig. 3D). Additionally, GlcNAc-6P positively correlated with the level of LDL-c (*P* < 0.05). In contrast, the level of fecal LPC 24: 1 was negatively correlated with CHOL and LDL-c (*P* < 0.05). The level of PC (14:1e/10:0) was positively correlated with HDL-c (*P* < 0.05), and PC (16:0/17:12) was negatively correlated with SBP (*P* < 0.05 and *P_fdr_* < 0.05). However, the trans-delta2-11-methyl-dodecenoic acid, DPPC, and 4-phenyl-3-buten-2-one showed no significant correlation with any of the MS clinical indicators.

### The association network among the microbiota, metabolites, and clinical parameters.

**FIG 4:**
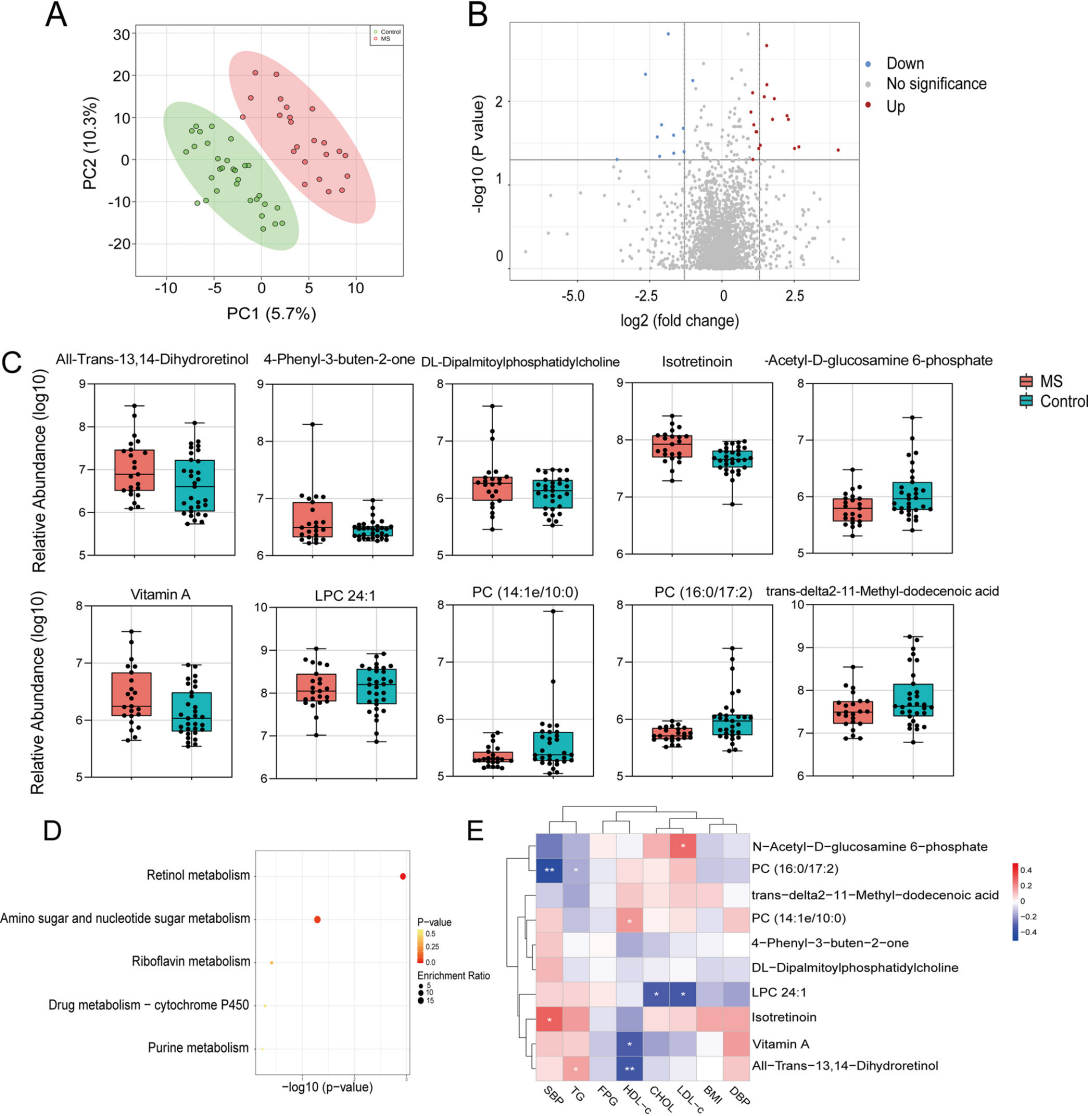
Metabolomics analysis between MS children and controls. (A) PLS-DA score plots of metabolic profiling in the MS and control groups. (B) Volcano plot of the altered metabolites. (C) Alteration of the top 10 metabolites. (D) The bubble plot of KEGG pathway enrichment analysis. (E) Correlation heat map analysis between the altered metabolites and clinical indicators. Red represents a positive correlation, and blue represents a negative correlation. *, *P* < 0.05; **, *P* < 0.01.

To further gain insight into the associations of MS-linked microbiota, altered metabolites, and clinical parameters, a Spearman correlation analysis was performed among the three data sets. A comprehensive network showing the relationships among the aforementioned omics (microbiome and metabolome) and clinical parameters was built. The top connected nodes are illustrated in Fig. 4A. Increased all-trans-13, 14-dihydroretinol was positively correlated with higher levels of *Lachnoclostridium* abundance as well as TG, CHOL, and LDL-C levels (*P* < 0.05) (Fig. 4A and B). Increased 4-phenyl-3-buten-2-one was positively correlated with the abundance of *Bacteroides* and negatively correlated with the abundance of *Parabacteroides* (Fig. 4A and C). Similarity, increased DPPC was positively correlated with the abundance of both *Dialiste*r and *Parabacteroides* (Fig. 4A and D). Interestingly, decreased PC (14:1e/10:0) and PC (16:0/17:12) were both positively correlated with increased *Parabacteroides* (Fig. 4A and E). Although many of the results did not withstand multiple testing correction, possibly due to the small sample size of each group, the data still helped to identify and integrate novel relationships among altered microbiota, metabolites, and host phenotypes.

### Biological function validation of candidate microbial metabolites.

**FIG 5:**
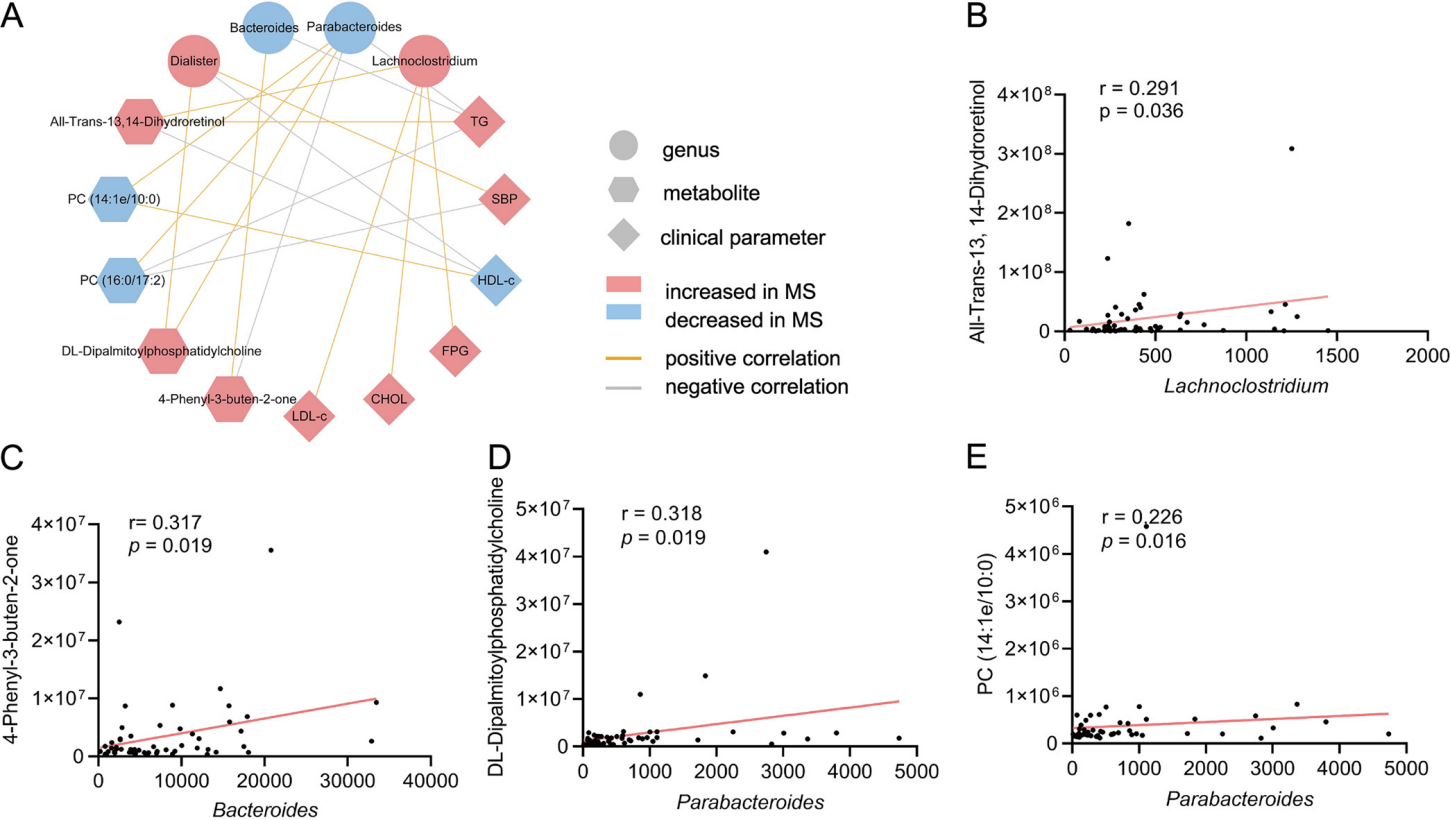
Associations among significantly altered microbiota, metabolites, and clinical indicators. (A) The association network among altered microbiota, metabolites, and MS clinical indicators. (B) The associations of all-trans-13, 14-dihydroretinol, and *Lachnoclostridium.* (C) The associations of DPPC and *Dialiste*r. (D) The associations of 4-phenyl-3-buten-2-one and *Bacteroides.* (E) The associations of 4-phenyl-3-buten-2-one and *Parabacteroides*.

Fig. 4 shows the main associations among altered microbiota, metabolites, and clinical indicators between the MS and control groups. To explore the pathogenic roles of microbial-associated metabolites in MS development, the top metabolites with heightened levels in the MS group, including all-trans-13, 14-dihydroretinol, DPPC, and 4-phenyl-3-buten-2-one, were selected for further bio-functional validation. HepG2 cells were exposed to different concentrations of the candidate metabolites for 24 h. All of these three candidates caused severe cytotoxicity in a dose-dependent manner (Fig. 5A). Moreover, the all-trans-13, 14-dihydroretinol could significantly influence the concentration of TG and CHOL, whereas DPPC and 4-phenyl-3-buten-2-one showed no effect on these lipid metabolic indicators (Fig. 5B; Fig. S5).

**FIG 6:**
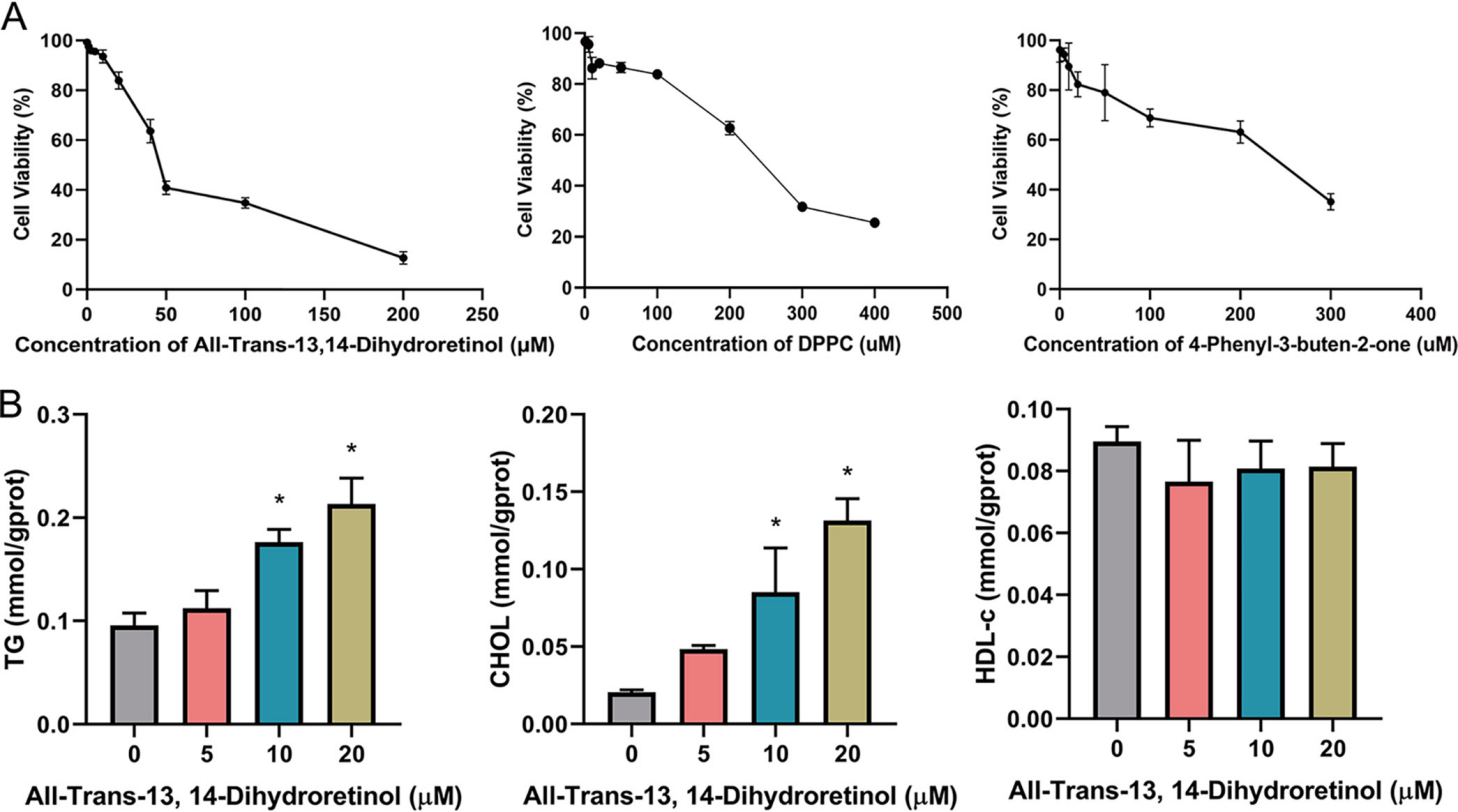
Validation of the role of candidate metabolites *in vitro*. (A) Inhibition of all-trans-13, 14-dihydroretinol, DPPC, and 4-phenyl-3-buten-2-one on the growth of HepG2 cells. (B) Effects of all-trans-13, 14-dihydroretinol (0, 5, 10, 20 μM) on intracellular TG, CHOL, and HDL-c production. Values are presented as the mean ± SD. *, *P* < 0.05.

To determine the molecular mechanism of these metabolites in lipid metabolism, we measured the gene expression profiles that were affected by the candidate metabolites. The effects of all-trans-13,14-dihydroretinol on the expression of lipid metabolism genes were determined, and they are described in Fig. 6. With increased concentrations of all-trans-13,14-dihydroretinol, the relative mRNA levels of lipid synthesis genes, including (*ACC1*, *SREBP1*, *SCD,* and *ACOX*) were upregulated accordingly. Besides, the relative mRNA level of inflammatory cytokines 6 (*IL-6*) was also increased when the concentration of all-trans-13,14-dihydroretinol increased. Moreover, compared with the control group, the relative mRNA level of the lipid oxidation gene (*PPARα*) decreased when the concentration of metabolite increased. However, the DPPC and 4-phenyl-3-buten-2-one showed limited gene regulation effects, with only one lipid metabolic gene being upregulated at a certain concentration level (Fig. S6 and S7). These results indicated that all-trans-13,14-dihydroretinol promoted lipid accumulation and inflammation in HepG2 cells.

## DISCUSSION

In this study, we performed a novel exploration of an integrated multiomics analysis of gut microbiome, metabolome, and clinical indicators in MS children and obese controls. The microbial and metabolic signatures of the MS children were significantly different from those of the obese controls. We identified 6 predominant genera and 3 elevated microbial metabolites that are closely related to MS in children. Furthermore, the biological functions of the elevated MS-associated microbial metabolites were validated *in vitro*. Finally, we found that the new biomarker (the all-trans-13,14-dihydroretinol) belongs to the retinol metabolism pathway and could significantly increase lipid accumulation in cells. These results imply that the microbial metabolites that are involved in retinol metabolism might be a potential driving factor for MS development.

In this study, the genera *Lachnoclostridium*, *Anaerostipes*, and *Dialister* were significantly enriched in the MS children, compared to the obese controls, whereas *Bacteroides*, *Subdoligranulum*, and *Parabacteroides* were decreased. LEfSe analyses have been used to find biomarkers that represent statistically and biologically consistent differences with statistical differences between groups in many studies (16, 26, 27). Thus, our results suggest these genera as potential biomarkers for obese children with MS. However, our results were not always consistent with those of previous studies, which typically used normal weight individuals as controls. Gallardo-Becerra et al. (15) investigated the association of gut microbiota and MS in children and reported that the genus *Collinsella* was significantly more abundant in the Mexican children with MS, whereas the genus *Porphyromonas* was decreased in the MS groups, compared to the normal weight children. The heterogeneity of the results is probably due to the difference in race, the selection of controls, and the relatively limited studies on children. Thus, it was meaningful to study obesity separately from MS, which allowed the researchers to obtain some new microbial biomarkers. Notably, the partially altered genera in our study were also consistent with the results of studies that analyzed other metabolic diseases (28–30). These findings indicated that gut dysbiosis existed in children with MS (31). In addition, in the alpha-diversity analysis, the observed index was used to measure the amount of unique OTUs found in each sample, the ACE and Chao indices reflected the community richness, and the Shannon and Simpson indices were used to assess the microbiota diversity. Although the diversity of the gut microbiota were not significantly different between the MS group and the control group, the rarefaction curve tended to be flat, and the amount of sequencing data were gradually reasonable, which suggested that bias of the sequence depth might not exist in the actual results. Also, a previous study reported that there were no significant differences in diversity between obese and MS subjects (16). Moreover, there were no statistically significant differences in terms of diversity, but there were significant differences in the abundance of genera between the two groups, and these gut microbial signatures may facilitate further mechanistic studies of MS and related diseases. Furthermore, the relative abundance of some identified genera was closely associated with at least one of the MS clinical indicators (32–34). In this work, the abundance of *Lachnoclostridium* was positively correlated with TG, LDL-c, and FPG levels, which was consistent with the results of a previous study (33). Meanwhile, we also found that the abundance of *Bacteroides* and *Parabacteroides* was negatively correlated with the serum TG level, with *Bacteroides* and *Parabacteroides* having been reported as potential probiotics in metabolic disease in some studies (35, 36). In addition, the correlation between *Dialister* and SBP was first revealed in this study, and we suggested an adverse impact of *Dialister*. Although the real physiological correlations and the underlying mechanism of gut microbiota with MS development need further confirmation, our findings suggested that the alterations in these bacterial communities may play an important role in the development of MS.

In addition to the analysis of MS-associated microbiota, we also conducted a metabolomics analysis which allowed us to explore the molecular pathways and identify key features associated with the progression of MS. In our study, the processes of retinol metabolism with all-trans-13,14-dihydroretinol were significantly increased in the MS group. It has been previously reported that retinol metabolism was closely correlated with MS in adults (37), and the elevated level of all-trans-13,14-dihydroretinol was also observed in other diseases, including atherosclerosis and cancer (38, 39). All-trans-13,14-dihydroretinol was a metabolite of retinol that was produced by retinol saturase (RetSat) (40, 41). We also observed that elevated all-trans-13,14-dihydroretinol was associated with typical MS indicators, including TG and HDL-c. These findings suggest that the metabolites that are involved in the retinol metabolism pathway enriched in the MS groups and that they probably contributed to the progression of MS. In addition, we found that the amino sugar and nucleotide sugar metabolism pathway was also enriched in the MS group, with the elevation of glucosamine-6-phosphate (GlcN6P) being positively correlated with serum LDL-c. Similarly, GlcN6P acted as a gatekeeper in the glycolysis metabolic pathway in previous studies (42). Other phospholipid metabolites, such as LPC 24: 1, PC (14:1e/10:0), and PC (16:0/17:12) were negatively correlated with the metabolic disease, and these results have been previously reported (43, 44). The pathways and pathway-associated metabolites might take part in the development of MS. These results demonstrated the potential effects of gut metabolites and provided further impetus for mechanistic studies on the occurrence and development of MS.

The metabolites associated with the progression of MS probably resulted from alterations of the gut microbiota. We further analyzed the integrative data of the microbiome, metabolome, and clinical indicators in the MS and control groups. As shown in the network, the elevated all-trans-13,14-dihydroretinol, DPPC, and 4-phenyl-3-buten-2-one that were enriched in the MS group were significantly correlated with MS-associated microbiota. All-trans-13,14-dihydroretinol was correlated with both *Lachnoclostridium* and the MS-related indicators. Notably, one recent study also found that retinol was affected by the abundance of *Lachnoclostridium* in MS patients (37). Moreover, the production and metabolism of all-trans-13,14-dihydroretinol in the host require the gut microbiota (45), suggesting that all-trans-13,14-dihydroretinol was closely associated with the gut microbiota. In addition, although DPPC and 4-phenyl-3-buten-2-one have previously been detected in biospecimens (46), limited information has been reported about DPPC and 4-phenyl-3-buten-2-one in human diseases and about their crosstalk with gut microbiota. These findings suggest that the microbial-associated metabolites may have important physiological significance in the occurrence and development of MS, although further research is needed to confirm the underlying mechanisms.

Although many of the associations did not withstand the multiple testing correction, possibly due to the small sample size of each group, the data still help to identify novel relationships among altered microbiota, metabolites, and clinical parameters. Moreover, in this work, the associations between the altered metabolites and MS were validated *in vitro* via cell experimentation. The gut microbiota is regarded as a metabolic organ that regulates its host metabolism, and it contacts other related organs through blood vessels and lymphatics (47). The liver is directly exposed to the gut metabolites through the portal vein, and it is the center of the homeostasis of global metabolism (48). Therefore, the HepG2 cell line was selected to conduct experiments for the validation of the biological functions of the candidate metabolites. The roles of the elevated microbial-metabolites, including all-trans-13,14-dihydroretinol, DPPC, and 4-phenyl-3-buten-2-one, were investigated for the first time. As anticipated, all three of the metabolites showed a dose-dependent toxicity to cell growth. The cytotoxicity of all-trans-13,14-dihydroretinol is probably due to it accelerating apoptosis in cells (49). However, only all-trans-13,14-dihydroretinol greatly induced the expression of genes that are involved in lipid synthesis and inflammation, and it inhibited the expression of lipid oxidation genes in HepG2 cells, which resulted in higher levels of TG and CHOL. The proteins involved in the retinol pathway, such as retinol binding protein 4 (RBP4), have been shown to stimulate hepatic SREBP1 and enhance lipogenesis through the PPARγ-dependent pathway (50). However, the role of all-trans-13,14-dihydroretinol in lipid synthesis was demonstrated for the first time in this study. Thus, all-trans-13,14-dihydroretinol might be a new biomarker for children with MS. Further studies are needed to illustrate the roles of these microbial-associated metabolites in the pathogenesis of MS, especially in children. In summary, the pivotal role of all-trans-13,14-dihydroretinol, in terms of its relation to the retinol metabolism pathway and contribution to the development of MS via its promotion of lipid synthesis, was uncovered for the first time.

This study has some advantages. We conducted a case-control study and a validation cell experimental study to explore the cause of MS. The association network among gut microbiota, metabolites, and host metabolism was illustrated via a multiomics analysis, which highlighted the crucial microbial-associated factors that contributed to the progression of MS. Moreover, we demonstrated that the expression of genes related to lipid metabolism was influenced by a crucial microbial metabolite *in vitro*, which confirmed the contribution of the metabolites in MS development. Besides the advantages mentioned above, we also acknowledge the following limitations. This was a single-center study that lacked a validation cohort, which might restrict the generalizability of the results. Due to the limited information collected regarding the subjects in this study, adjustments for potential confounding factors via multivariate mathematical models were not performed, warranting additional investigations that involve larger prospective cohorts and more information to verify our results. Moreover, using 16S rRNA amplicon sequencing rather than deep metagenomics sequencing limited functional inference in the bacterial strains. To overcome the shortage, the fecal metabolome was profiled as a functional readout of the gut microbiome (51), but whether these metabolites were derived from gut microbiota needs to be clarified in the future. Therefore, larger, multi-center, extensively functional studies, both *in vitro* and *in vivo*, as well as molecular multiomics technology, are necessary to explore the molecular mechanisms of gut microbiota in the development of MS.

## MATERIALS AND METHODS

### Study population and data collection.

**FIG 7:**
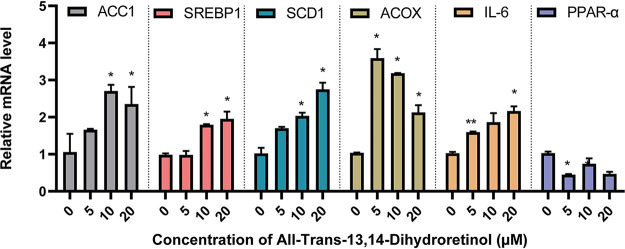
Lipid metabolic gene expression profiles affected by all-trans-13, 14-dihydroretino *in vitro*. Values are presented as the mean ± SD. *, *P* < 0.05; **, *P* < 0.01.

The overview of this study design is summarized in Fig. 7. For this case-control study, obese children between 10 and 18 of age years were recruited from the Institute of Child Health, Hunan Children’s Hospital (Hunan, China) during June and December of 2019. Obese children were screened using BMI-for-age and BMI-for-sex, according to national standards (52). MS was diagnosed according to the Pediatrics Branch of the Chinese Medical Society Guidelines (53). The controls were simple obese children. To exclude the potential effects of potential confounding factors on the microbiome analysis, children were excluded if they had a history of chronic inflammatory bowel disease, drugs, or antibiotics used within 3 months. The Xiangya School of Public Health Central South University Ethics Research Committee approved the study (XYGW-2018-04), which was conducted in accordance with the Declaration of Helsinki. Written informed consent was obtained from parents of the enrolled subjects. Demographic and clinical parameters of the participants were collected from questionnaires as well as from electronic medical records from hospitals. The details of the measurement methods used are described in the supplemental material.

### DNA extraction, 16S rRNA gene amplicon sequencing, and data processing.

Fecal microbiota genomic DNA was isolated using a TIANGEN DNA Isolation Kit (Tiangen, Beijing, China). Briefly, genomic DNA was extracted using 200 mg fecal material. Then the specific lysis buffer and protease K from the kit were added for mechanical cell lysis (0.25 g bead-beating) at 60 Hz for 1 min. The V4 regions of the 16S rRNA gene were amplified using the universal bacteria primer pair F515 (5′-GTGCCAGCMGCCGCGGTAA-3′) and R806 (5′-GGACTACVSGGGTATCTAAT-3′) (54), and this was performed on an Illumina MiSeq platform at the Novogene (NovaSeq 6000, Beijing, China) (55). After undergoing quality control via Qiime (version 1.9), the raw sequences with similarity values of ≥97% were grouped into operational taxonomic units (OTUs). Each OTU was taxonomically assigned via the Greengenes database (version 13.8). The OTU information was then normalized by the 16S rRNA gene copy numbers, and then the compositions and abundance of microbiota were inferred for each sample (56).

### Microbiome analysis.

The alpha and beta diversity values of the microbiome analyses were performed using MicrobiomeAnalyst (https://www.microbiomeanalyst.ca/). All of the analyses in MicrobiomeAnalyst were performed on rarefied data. More specifically, the alpha diversity index at each sampling used diversity alpha-rarefaction with the default parameters. The step length was set as 10, and the alpha diversity index was calculated for all samples in a sparse table with 10 iterations. To measure the alpha diversity, we performed nonparametric Mann-Whitney-Wilcoxon tests for the differences of numbers of observed features, including the Chao and ACE indices (richness) and the Shannon and Simpson indices (evenness and richness). To elevate the beta diversity, we conducted a permutational multivariate analysis of variance (PERMANOVA) test for the differences of the Bray-Curtis distance, and the unweighted and weighted UniFrac distances, respectively, between the two groups (57, 58). A principal coordinates analysis (PCoA) was conducted to explore and visualize the similarities and differences of the microbiota between the two comparison groups (59). A LEfSe analysis (http://huttenhower.sph.harvard.edu/lefse) (LDA score [log_10_] = 3 as the cutoff value) was performed to analyze the predominance of genera between groups (60). Nonparametric Mann-Whitney-Wilcoxon tests were used to reconfirm the differential abundance of gut microbiota between obese and MS children. Tax4Fun was used for predicting functional abundances, including KEGG orthologs and pathways, based on marker gene sequences (61). A stamp analysis was used to test the different functions and pathways of microbiota in the MS children and in the obese controls. A Spearman analysis and a correlation heat map were used to evaluate the associations between the altered metabolites and the clinical indicators.

### Metabolite extraction and UPLC-MS/MS analysis.

Fecal samples were collected for nontargeted metabolite profiling. Metabolites were extracted via homogenization and sonication with acetonitrile/methanol/water (2:2:1, vol/vol/vol) containing an isotopically labeled internal standard mixture. The same volume of metabolites was extracted from each sample and was mixed as a quality control (QC) sample. The extracted and mixed metabolites were transferred to a fresh glass vial for an ultra-performance liquid chromatography-tandem mass spectrometry analysis (UPLC-MS/MS) with positive and negative ion-modes electrospray ionization. The metabolites were identified and quantified using matching databases (mzCloud, mzVault, and MassList), based on the retention time and the mass-to-charge ratio (*m/z*). The relative quantity of each metabolite was determined by comparing its peak areas to the total peak area. After background ion removal with a blank sample and data normalization, the metabolome data were obtained.

### Metabolome analysis.

The metabolomics data analysis was conducted using R and online versions of MetaboAnalyst (http://www.metaboanalyst.ca). The metabolome data were log_2_ transformed and scaled to unit variance in a multivariate analysis. Prior to the following metabolome analyses, a principal components analysis was performed to filter out samples outside of the 95% CI. A supervised model of partial least-squares discrimination analysis (PLS-DA) was applied to assess the metabolic alterations among groups in Simca-P V.14.0 (Umetrics AB) (62). A nonparametric univariate method (the Wilcoxon rank-sum test) and variable importance in projection (VIP) scores from a pairwise PLS-DA analysis were performed to identify significantly altered metabolites. Metabolites with (i) VIP scores of >2, (ii) fold changes of >2.0 or <0.5, and (iii) *P_fdr_* < 0.05 were regarded as significantly altered. A Volcano plot was drawn to display an overview of the alteration of the metabolites using the R package ggplot2. A permutation test was performed 200 times to assess the risk of overfitting for the PLS-DA model, and only confidently annotated metabolites were selected for the differential analysis. A pathway enrichment analysis was conducted using the KEGG pathway database. A Spearman analysis and a correlation heat map were used to evaluate associations between the altered metabolites and the clinical indicators.

### Candidate metabolites treatment in HepG2 cells.

HepG2 cells were obtained from the Cancer Research Institute of Central South University (Changsha, China) and were cultured in high-glucose Dulbecco’s Modified Eagle’s Medium (DMEM) (Gibco BRL, Grand Island, New York, USA) supplemented with 10% (vol/vol) fetal bovine serum and 1% penicillin/streptomycin at 37°C in a humidified atmosphere of 5% CO_2_. A 96-well plate with 5,000 cells was seeded and incubated at 37°C. Then, the candidate metabolites stock solution was added to the cell culture media for 24 h. A CCK8 assay (Vazyme, Nanjing, China) was performed to assess the impact on cell viability. HepG2 cells were treated with candidate metabolites via incubation in a 6-well plate for 24 h. The intracellular total triglycerides (TG), total cholesterol (CHOL), and high-density lipoprotein cholesterol (HDL-c) levels were measured using enzymatic reagent kits (Jiancheng, Nanjing, China), according to the manufacturer’s instructions. On the other hand, cells were washed with phosphate buffered solution (PBS, pH 7.0) and collected after treatment for RNA isolation. All experimental conditions were performed in three biological replicates. Total RNA was extracted and purified using TRIzol reagent (Vazyme, Nanjing, China), following the manufacturer’s protocol. The cDNA was synthesized using HiScript II Q RT SuperNix for qPCR (+gDNA wiper) (Vazyme, Nanjing, China). The primers used in this study are shown in Table S4. The gene expression procedures were assessed using a LightCycler 480 real-time PCR system (Roche Diagnostics) with SYBR technology. The relative quantification values for each mRNA were calculated via the 2^−△△Ct^ method.

### Statistical analysis.

Clinical data were expressed as the mean ± standard deviation (SD). The differences in clinical parameters between the MS group and the control group were evaluated via *t* tests. All of the statistical tests were performed using SPSS (version 24.0, IBM Corp., USA) and GraphPad Prism (version 8, San Diego, CA, USA). A Spearman correlation test and a correlation heatmap were used to evaluate the associations among the altered microbiota, metabolites, and clinical indicators. The network was visualized using Cytoscape (version 3.9.0). For the microbiome and metabolome analyses, multiple hypothesis tests were adjusted using the Benjamini-Hochberg false discovery rate (FDR) correction, and a *P_fdr_* value of <0.05 was considered to be indicative of a statistically significant result. In addition, two-tailed Student’s *t* tests were performed to assess the differences in the lipid indices and gene expression of culture cells between the treatment and control groups. *P* values of <0.05 were to be indicative of statistically significant results.

### Conclusions.

In conclusion, this study represented the first endeavor to comprehensively analyze gut microbial and metabolic features in Chinese children with MS. We identified 6 predominant genera and 3 elevated microbial metabolites that were closely related to MS in children. The associations among altered microbiota, metabolites, and MS clinical indicators were revealed. *In vitro* experiments suggested that the all-trans-13, 14-dihydroretinol could significantly activate lipid synthesis and inflammation. All-trans-13, 14-dihydroretinol might be a new biomarker that contributes to MS in children. These findings provided new insights with which to develop novel and effective therapeutic strategies for metabolic diseases.

### Ethics approval and consent to participate.

The studies involving human participants were reviewed and approved by the Xiangya School of Public Health Central South University Ethics Research Committee. Written informed consent to participate in this study was provided by the legal guardian/next of kin of each participant.

### Data availability.

All of the sequencing data that support the findings of the study are openly available in the National Center for Biotechnology Information (NCBI) via the accession number PRJNA838778.
