# Supplementary material for: Identifying the Novel Gut Microbial Metabolite Contributing to Metabolic Syndrome in Children Based on Integrative Analyses of Microbiome-Metabolome Signatures
Source: Microbiol Spectr. 2023 Feb 16;11(2):e03771-22. doi: 10.1128/spectrum.03771-22 (PMC10101147; doi:10.1128/spectrum.03771-22)
Supplement: Supplemental file 1 — Supplemental material. Download spectrum.03771-22-s0001.pdf, PDF file, 0.6 MB [file spectrum.03771-22-s0001.pdf]

Supplementary material for

Identifying the novel gut microbial metabolite contributing to metabolic syndrome in children based on integrative analyses of microbiome-metabolome signatures

Jia Wei [1,2], Wen Dai [1,2], Xiongfeng Pan [1,2], Yan Zhong [3], Ningan Xu [3], Ping Ye [1,2], Jie Wang [1,2], Jina Li [1,2], Fei Yang [1,2,4], Jiayou Luo [1,2]*, Miyang Luo[1,2]*

[1]Xiangya School of Public Health, Central South University, Changsha, 410078, Hunan, China.

[2]Hunan Provincial Key Laboratory of Clinical Epidemiology, Central South University, Changsha, 410078, Hunan, China

[3]Institute of Children Health, Hunan Children's Hospital, Changsha 410007, Hunan, China.

[4]Hunan Province Key Laboratory of Typical Environmental Pollution and Health Hazards, School of Public Health, University of South China, Hengyang, 421001, Hunan, China

*Corresponding author: Jiayou Luo and Miyang Luo

Xiangya School of Public Health, Central South University, Changsha, 410078, Hunan, China.

E-mail: jiayouluo@126.com (J.L.); miyangluo@csu.edu.cn (M.L.)

Running Title: Microbial signatures and metabolic syndrome

Supplementary Methods

Diagnostic criteria of MS

MS must be central obesity children (the waist circumference higher than the 90th percentile value of the same age. Besides, the presence of two or more of these components below: (1) hyperglycemia: fasting blood glucose ≥5.6 mmol L^{-1}; 2 hour glucose tolerance test glucose ≥ 7.8 mmol L^{-1}, but < 11.1 mmol L^{-1}; or type 2 diabetes; (2) hypertension: systolic blood pressure ≥ 90% or diastolic blood pressure ≥ 95 % that of children in the same age and sex. (4) low high-density cholesterols (HDL-C < 1.03 mmol L^{-1}) or high non-high-density lipoprotein cholesterol (non-HDL-C ≥ 3.76 mmol L^{-1}); (5) hypertriglyceridemia: serum triglyceride (TG) ≥1.47mmol L^{-1}.

Anthropometric, Demographic and Clinical Laboratory Measurements

Clinical parameters including weight, height, blood pressure (BP), waist circumference (WC), Hip circumference (HC) were measured by trained nurses using standard protocols and calibrated instruments. Body fat were assessed using bio-impedance (SHHC Body Composition Analysis, China). BMI was calculated as weight (in kilograms) divided by square of height (in meters). Fasting venous blood samples were collected from the veins of the subjects after 12 h of fasting. The laboratory session including triglycerides (TG), cholesterol (CHOL), low-density cholesterol (LDL), high-density cholesterol (HDL), glucose (GLU), aspartate aminotransferase (AST), alanine aminotransferase (ALT), serum C-peptide and insulin, etc. were were measured using an auto analyzer (Olympus AU5400, Tokyo, Japan) (Table S1).

Supplementary Results

Table S1. Characteristics of MS patients and obese children subjects

Anthropometric and Demographic	MS (n=23)	Control (n=31)	P-value
Female gender (%)	95.65	96.77	0.652
Age (year)	13.87±1.914	13.26±1.39	0.520
BMI (kg/m^2)	28.84±3.68	28.54±3.48	0.900
Weight (kg)	69.99±16.75	66.03±15.26	0.817
Height (m)	154.87±10.14	152.95±10.17	0.494
Neckline (cm)	35.82±3.43	34.68±2.42	0.159

Waistline (cm)	92.88±10.09	93.31±9.99	0.876
Hipline (cm)	98.94±9.22	96.28±18.31	0.526
Body fat mass (kg)	26.14±6.61	26.80±8.51	0.295
Lean body mass (kg)	43.60±11.50	40.12±8.35	0.213
Percentage of body fat (%)	37.46±4.78	39.77±6.12	0.600
Visceral fat (cm^2)	121.31±28.80	131.64±40.93	0.163
Muscle mass (kg)	40.49±11.75	37.78±7.82	0.314
Basal metabolic rate (kcal)	1318.61±244.85	1244.29±179.37	0.203
SBP (mmHg)	128.31±9.97	120.23±11.61	0.018*
DBP (mmHg)	71.87±11.08	72.71±11.67	0.790
Laboratory examination			
TG (mmol/L)	2.09±0.97	1.06±0.27	<0.001*
CHOL (mmol/L)	4.24±0.23	3.71±0.24	0.012*
HDL-c (mmol/L)	1.03±0.23	1.22±0.152	0.001*
LDL-c (mmol/L)	2.33±0.75	2.092±0.62	0.381
FPG (mmol/L)	4.77±1.89	4.32±0.41	0.084
Insulin (μU/mL)	35.72±20.78	29.87±13.43	0.115
C-peptide (ng/mL)	3.99±1.60	3.60±1.03	0.073
AST (IU/L)	31.89±18.33	28.82±16.20	0.518
ALT (IU/L)	57.43±10.34	52.64±49.34	0.706
AST/ALT	0.62±0.15	0.73±0.30	0.007*
TBAC (μmol/L)	4.49±4.33	3.45±1.69	0.229
TBIL (μmol/L)	11.16±3.29	11.52±5.90	0.793
DBIL (μmol/L)	3.78±1.35	4.03±2.13	0.633
IBIL (μmol/L)	7.37±2.33	9.03±9.07	0.397
ALB (g/L)	42.92±1.90	41.23±2.40	0.106
GLO (g/L)	29.49±2.45	29.26±3.02	0.118
A/G	1.47±0.14	1.42±0.17	0.350

Values are expressed as means ± SD. BMI: body mass index; SBP: systolic blood pressure; DBP: diastolic blood pressure; TG: triglycerides; CHOL: total cholesterol; HDL-c: high-density lipoprotein cholesterol; LDL-c: low-density lipoprotein cholesterol; FPG: fasting plasma glucose; AST: aspartate aminotransferase; ALT: alanine aminotransferase; TBAC: total bile acid; TBIL: total bilirubin; DBIL: direct bilirubin; IBIL: indirect bilirubin; ALB: albumin; GLO: globulin; A/G: albumin/globulin. The t-test was used to evaluate differences in anthropometric, demographic data and clinical Laboratory between the two groups. Unless otherwise stated, $p < 0\ .05$ was considered statistically significant.

Table S2. Significantly altered fecal metabolites in MS children and obese controls.

Metabolites	Fold-changed	*p*-value	VIP score
DMK	3.78	0.017	3.36
All-Trans-13,14-Dihydroretinol	3.38	0.039	2.08
YPH	3.33	0.013	2.97
DL-Dipalmitoylphosphatidylcholine	3.26	0.030	2.09
4-Phenyl-3-buten-2-one	3.07	0.032	3.10
Isotretinoin	3.05	0.011	3.10
Vitamin A	2.91	0.016	2.89
Galangin	2.82	0.007	3.25
Emodin	2.80	0.022	3.08
5-(tert-butyl)-2-methyl-N-(4-nitrophenyl)-3-furamide	2.75	0.041	2.60
5-[(E)-2-(3,5-dihydroxyphenyl)ethenyl]-2-methoxybenzene-1,3-diol	2.59	0.043	2.30
4-[2-(2-oxo-1-imidazolidinyl)ethyl]-1lambda~6~,4-thiazinane-1,1-dione	2.34	0.033	2.20
4-Hydroxyalprazolam	2.30	0.016	2.69
1-methyl-N-(3-methyl-5-cinnolinyl)-1H-imidazole-4-sulfonamide	2.28	0.026	2.69
2,3-di(acetyloxy)-3-cyano-1-[1,2-di(acetyloxy)ethyl]propyl acetate	2.15	0.005	2.99
Rhein	2.09	0.010	2.45
Kahweol	2.05	0.035	2.42
LPE 16:0	0.49	0.014	2.70
Aflatoxin G1	0.46	0.035	2.13
PC (20:3e/4:0)	0.36	0.048	2.52
N-Acetyl-D-glucosamine 6-phosphate	0.32	0.016	2.81
5-methoxy-8,8-dimethyl-2-phenyl-4H,8H-pyrano[2,3-h]chromen-4-one	0.28	0.002	3.20
Perillartine	0.28	0.014	2.69
trans-delta2-11-Methyl-dodecenoic acid	0.27	0.043	2.72
Indirubin	0.22	0.020	2.77
PC (16:0/17:2)	0.22	0.014	2.97
NVP-231	0.14	0.026	3.22
LPC 24:1	0.09	0.049	2.28
PC (14:1e/10:0)	0.08	0.039	2.42

Table S3. The list of significant associations among altered microbiota, metabolites and clinical indicators

Items	interacted with	Corr.	*p*-value	relation
All-Trans-13/14-Dihydroretinol	*Lachnoclostridium*	0.291	0.036	positive
All-Trans-13/14-Dihydroretinol	HDL-c	-0.311	0.022	negative
All-Trans-13/14-Dihydroretinol	TG	0.269	0.049	positive
Lachnoclostridium	CHOL	0.290	0.033	positive
Lachnoclostridium	FPG	0.225	0.049	positive
Lachnoclostridium	LDL-c	0.292	0.032	positive
Bacteroides	TG	-0.360	0.007	negative
DL-Dipalmitoylphosphatidylcholine	*Dialister*	0.398	0.003	positive
DL-Dipalmitoylphosphatidylcholine	*Parabacteroides*	0.318	4.53E-04	positive
Dialister	HDL-c	-0.286	0.036	negative
Dialister	SBP	0.301	0.026	positive
HDL-c	PC (14:1e/10:0)	0.327	0.015	positive
4-phenyl-3-buten-2-one	*Bacteroides*	0.317	0.019	positive
4-phenyl-3-buten-2-one	*Parabacteroides*	-0.288	0.019	negative
PC (14:1e/10:0)	*Parabacteroides*	0.226	0.016	positive
PC (16:0/17:2)	*Parabacteroides*	0.349	0.010	positive
Parabacteroides	TG	-0.279	0.041	negative
SBP	PC (16:0/17:2)	-0.456	5.38E-04	negative
TG	PC (16:0/17:2)	-0.270	0.048	negative

Table S4. The primers used in qRT-PCR analysis

Genes	Forward primer (5’--3’)	Revers primer (5’--3’)
ACC1	CTCTTGGCCTTTTCCCGGTC	GTTATCCCCAAACCCAGGCA
SREBP1	CGGGAGGATGGACTGACTT	GAGGAGGCTTCTTTGCTGTG
SCD1	TGCTTGGCAGCGGATAA	GGAGGACTGCGGTTTCG
ACOX	AATCAGGGCACCACTGCTC	CCAAGCCTCGAAGGTGAGTT
IL-6	CCTTCGGTCCAGTTGCCTTCTC	AGAGGTGAGTGGCTGTCTGTGT
PPAR-α	CATCACGGACACGCTTTC	CCCGCAGATTCTACATTCG

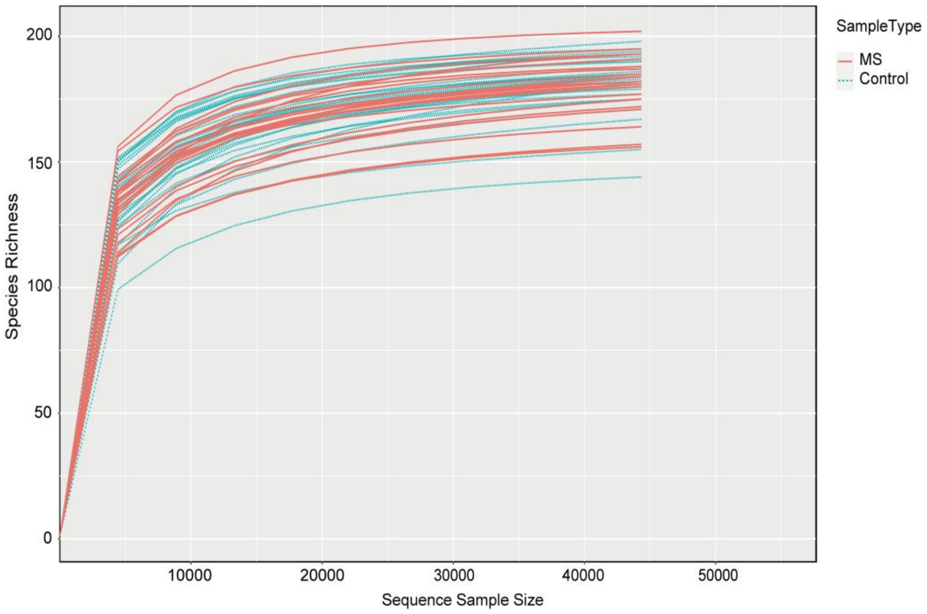

FIG S1 Rarefaction curve analysis of the species richness on each sample in different groups.

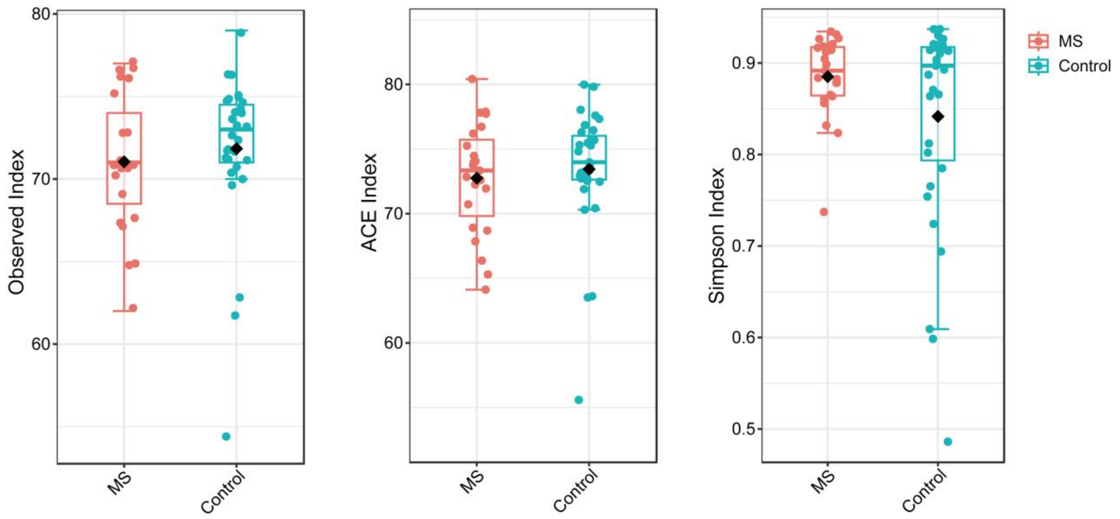

FIG S2 The alpha diversity (Observe, ACE, and Simpson index) of gut microbiota between the MS and the control group.

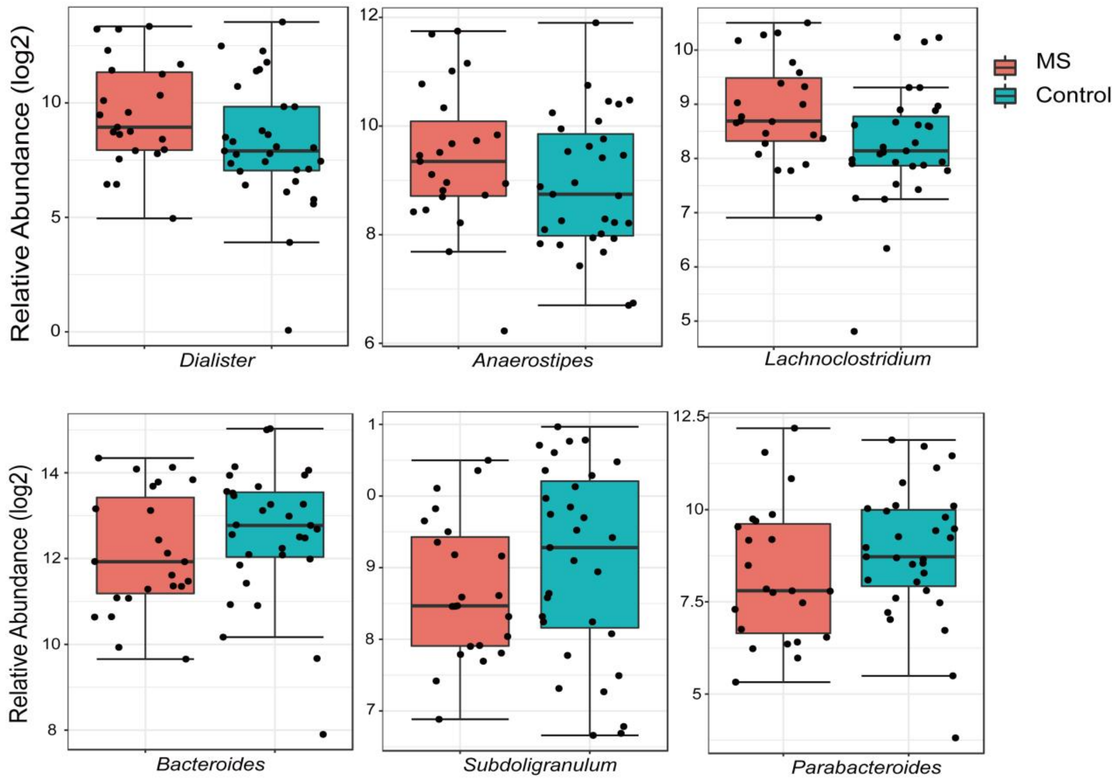

FIG S3 Significant difference fecal microbitota in genus level between MS and control group. MS: metabolic syndrome children; Control: obese children.

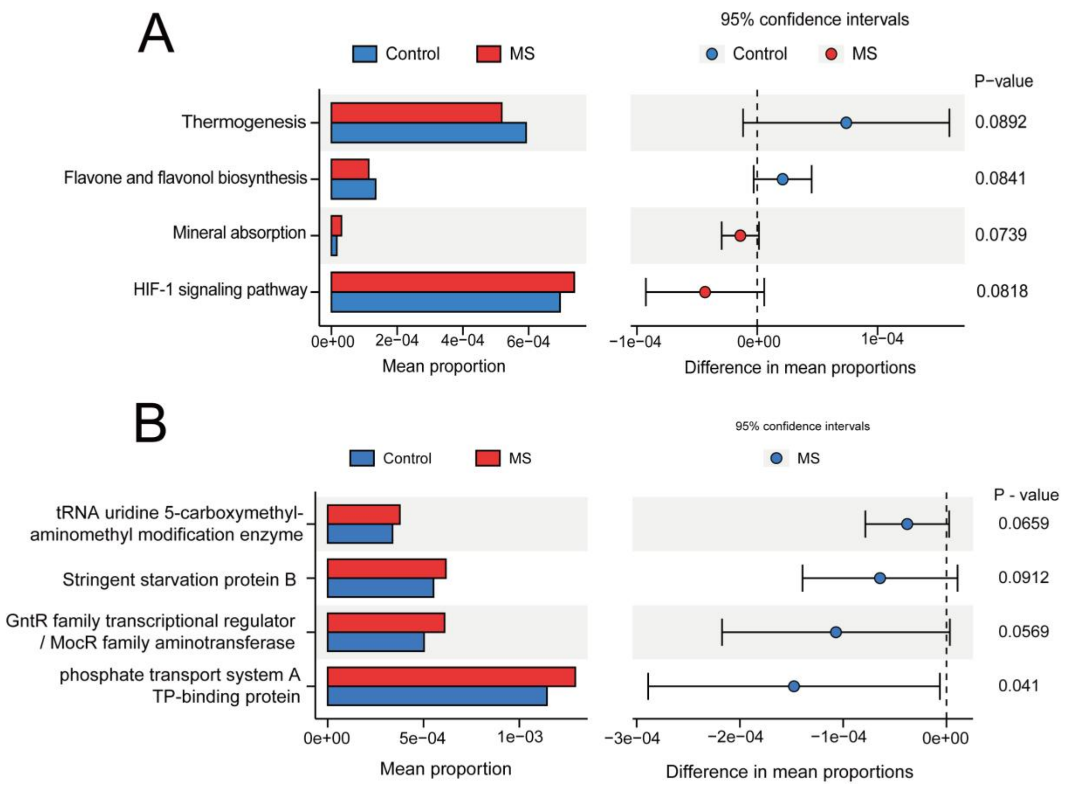

FIG S4 Functional prediction of fecal microbiota community. (A) Pathway prediction of fecal microbiota community in MS and control group. (B) Functional enzymes prediction of fecal microbiota community in MS and control group. Extended error bar plot for each pathway/function indicating differences in mean proportions for each pair of groups. $p < 0.05$ was considered statistically significant in the MS and control group.

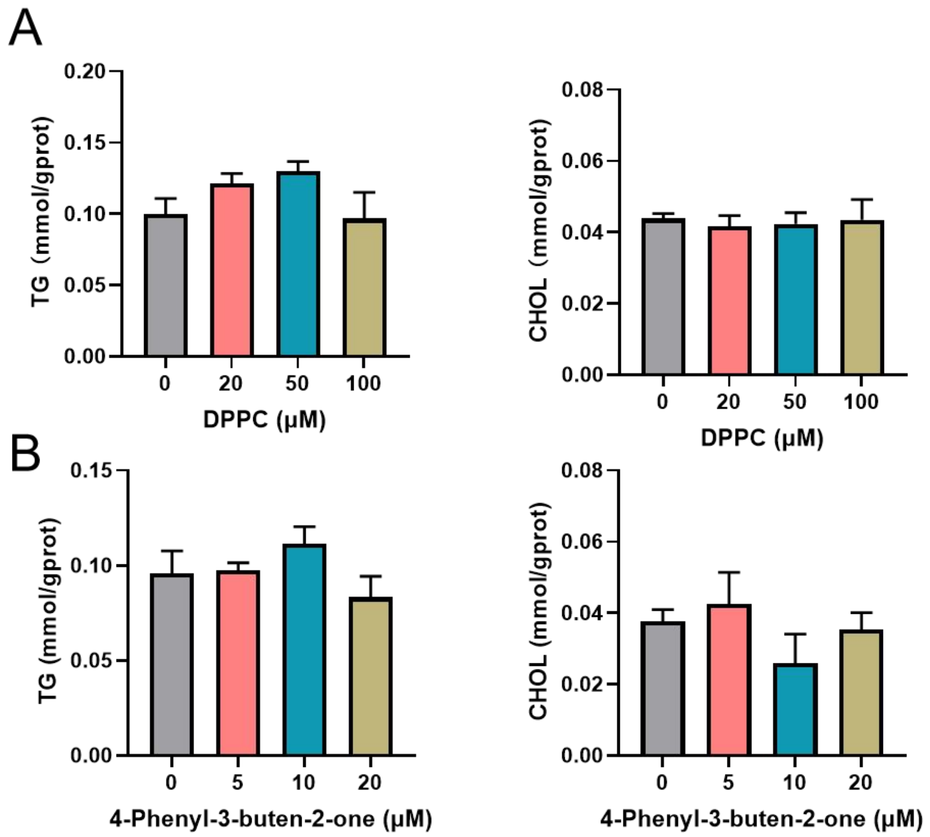

FIG S5 Validation the role of candidate metabolites in vitro. (A) Effects of DPPC (0, 20, 50, 100 μM) on intracellular TG, and CHOL production. (B) Effects of 4-phenyl-3-buten-2-one (0, 5, 10, 20 μM) on intracellular TG, and CHOL production.Values are presented as mean ± SD. *$p < 0.05$.

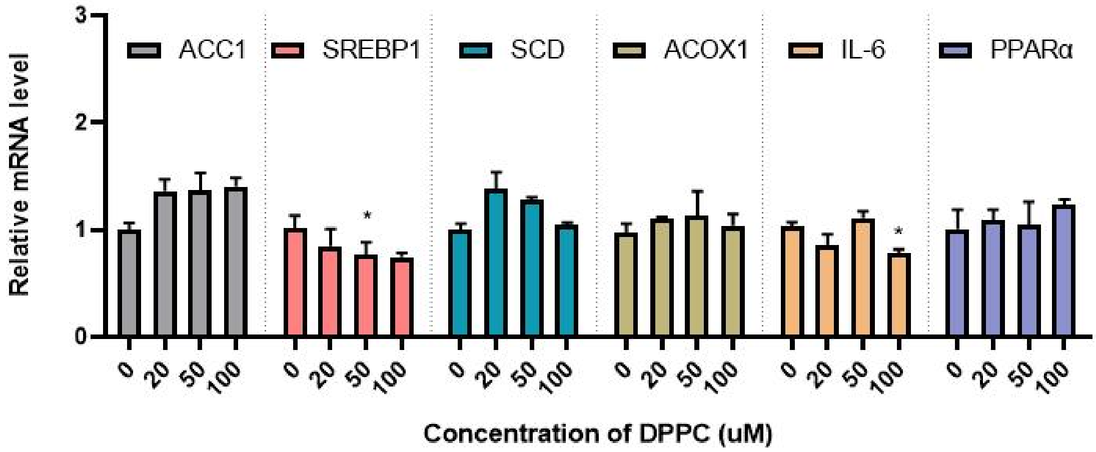

FIG S6 Lipid metabolic gene expression profiles effected by DPPC (0, 20, 50, 100 μM) in vitro. Values are presented as mean ± SD. *$p < 0.05$.

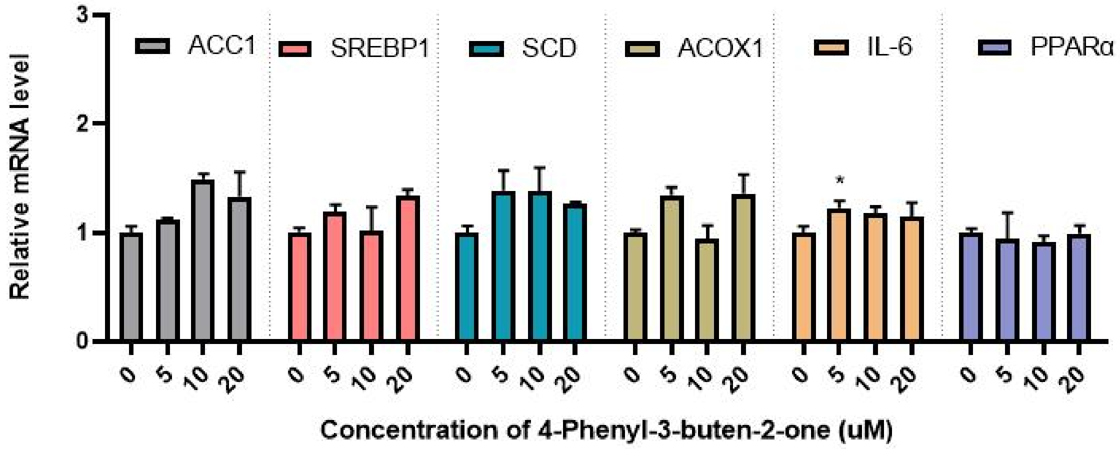

FIG S7 Lipid metabolic gene expression profiles effected by 4-Phenyl-3-one (0, 5, 10, 20 μM) in vitro. Values are presented as mean ± SD. *$p < 0.05$.
